# Computed tomography aortic valve calcium scoring for the assessment of aortic stenosis progression

**DOI:** 10.1136/heartjnl-2020-317125

**Published:** 2020-10-05

**Authors:** Mhairi Katrina Doris, William Jenkins, Philip Robson, Tania Pawade, Jack Patrick Andrews, Rong Bing, Timothy Cartlidge, Anoop Shah, Alice Pickering, Michelle Claire Williams, Zahi A Fayad, Audrey White, Edwin JR van Beek, David E Newby, Marc R Dweck

**Affiliations:** 1 The University of Edinburgh Centre for Cardiovascular Science, Edinburgh, UK; 2 Icahn School of Medicine at Mount Sinai BioMedical Engineering and Imaging Institute, New York, New York, USA; 3 Edinburgh Imaging, The University of Edinburgh-The Queen's Medical Research Institute, Edinburgh, UK

**Keywords:** aortic stenosis, cardiac computer tomographic (CT) imaging, echocardiography

## Abstract

**Objective:**

CT quantification of aortic valve calcification (CT-AVC) is useful in the assessment of aortic stenosis severity. Our objective was to assess its ability to track aortic stenosis progression compared with echocardiography.

**Methods:**

Subjects were recruited in two cohorts: (1) a *reproducibility cohort* where patients underwent repeat CT-AVC or echocardiography within 4 weeks and (2) a *disease progression cohort* where patients underwent annual CT-AVC and/or echocardiography. Cohen’s d-statistic (*d*) was computed from the ratio of annualised progression and measurement repeatability and used to estimate group sizes required to detect annualised changes in CT-AVC and echocardiography.

**Results:**

A total of 33 (age 71±8) and 81 participants (age 72±8) were recruited to the reproducibility and progression cohorts, respectively. Ten CT scans (16%) were excluded from the progression cohort due to non-diagnostic image quality. Scan-rescan reproducibility was excellent for CT-AVC (limits of agreement −12% to 10 %, intraclass correlation (ICC) 0.99), peak velocity (−7% to +17%; ICC 0.92) mean gradient (−25% to 27%, ICC 0.96) and dimensionless index (−11% to +15%; ICC 0.98). Repeat measurements of aortic valve area (AVA) were less reliable (−44% to +28%, ICC 0.85).

CT-AVC progressed by 152 (65–375) AU/year. For echocardiography, the median annual change in peak velocity was 0.1 (0.0–0.3) m/s/year, mean gradient 2 (0–4) mm Hg/year and AVA −0.1 (−0.2–0.0) cm^2^/year. Cohen’s d-statistic was more than double for CT-AVC (*d*=3.12) than each echocardiographic measure (peak velocity *d*=0.71; mean gradient *d*=0.66; AVA *d*=0.59, dimensionless index *d*=1.41).

**Conclusion:**

CT-AVC is reproducible and demonstrates larger increases over time normalised to measurement repeatability compared with echocardiographic measures.

## Introduction

Aortic stenosis represents a major cause of morbidity and mortality, the burden of which is set to increase. Currently, the only definitive treatment is surgical or transcatheter aortic valve replacement in patients with severe symptomatic stenosis. Aortic valve narrowing progresses inexorably but at a variable and unpredictable rate in individual patients. Frequent echocardiographic follow-up is therefore mandated to determine the optimal timing for intervention.^1^

The clinical assessment of aortic stenosis severity is based on two-dimensional echocardiography and Doppler, with measurement of the peak jet velocity, mean gradient and aortic valve area (AVA) frequently used to guide severity assessment.^1^ However, the measurement of disease progression by echocardiography is challenged by small changes in these markers of haemodynamic severity over time, combined with a relatively high degree of variability between measurements.^2 3^ A complementary imaging technique capable of providing improved reproducibility and sensitivity to change is therefore desirable. This is of importance in the clinical setting for accurate tracking of disease progression, and also in the research arena, where imaging end points are increasingly being used to assess the effects of novel therapies on aortic stenosis progression.

Quantification of aortic valve calcification by non-contrast CT (CT-AVC) has demonstrated promise in accurately defining the valvular calcification burden, with sex-specific thresholds demonstrating good diagnostic accuracy compared with concordant echocardiography and providing incremental prognostic information.^4 5^ In this study, our objective was to assess the ability of CT-AVC to monitor aortic stenosis progression compared with echocardiographic assessments. We investigated scan-rescan reproducibility and annual progression of both CT-AVC and echocardiographic measurements in a large prospective cohort of patients with aortic stenosis.

## Methods

### Study population

Participants aged >50 years attending the outpatient Department of the Edinburgh Heart Centre with aortic stenosis (peak aortic jet velocity >2 m/s) were recruited into two cohorts as part of a previously reported study (NCT01358513) and an ongoing clinical trial (NCT02132026).^6 7^ In the *reproducibility cohort*, participants underwent repeat echocardiography or CT-AVC scanning within 4 weeks. In the *disease progression cohort*, participants underwent either repeat echocardiography, CT or both after at least 1 year. This research was undertaken without patient involvement. Patients were not invited to comment on the study design and were not consulted to interpret the results or contribute to writing or editing of this document for readability or accuracy.

### Baseline assessment

All participants underwent a comprehensive baseline clinical assessment. Echocardiography was performed by an experienced echocardiographer (AW) using a prespecified protocol according to the European Society of Echocardiography guidelines^8^ on the same scanner of a British Society of Echocardiography accredited laboratory. Multiple acoustic windows were assessed with the S51 and D2cwc probes (Philips Medical Systems, The Netherlands). Aortic stenosis severity was assessed on the basis of the peak velocity, mean gradient, AVA (calculated using the continuity equation) and dimensionless index (DI; defined as left ventricular outflow tract (LVOT) peak velocity divided by aortic peak velocity) according to the American Heart Association and American College of Cardiology guidelines.^9^

To measure CT-AVC, an ECG-gated non-contrast CT scan was performed during inspiration on a 128 multidetector scanner (Biograph mCT Siemens, 40 mA/rot tube voltage 100 kV, tube current selected using automatic exposure control).^10^ In the absence of contraindications, participants were administered beta-blockade to achieve a resting heart rate ≤65 bpm. Images were reconstructed in the axial plane with 3 mm slice width and 1.5 mm increment. Valvular calcification was quantified by the Agatston method^11^ using dedicated analysis software (Vitrea Advanced, Vital Images, Minnetonka, USA; online supplemental figure 1). Care was taken to exclude calcium from extravalvular structures such as the mitral valve annulus and coronary arteries. When confluent calcium extended into the ascending aorta, the origin of the left coronary artery was set as the most rostral slice beyond which further calcium was excluded.^4 12^ The aortic valve calcium burden was expressed as CT-AVC in Agatston Units (AU).

10.1136/heartjnl-2020-317125.supp1 Supplementary data

### Scan-rescan reproducibility

The reproducibility cohort consisted of two groups of participants with aortic stenosis who underwent either repeat CT-AVC scoring or echocardiographic assessment of their valve. In one group, CT was performed at baseline and again within 4 weeks. Scan-rescan reproducibility was determined for CT-AVC measurements. In a second group, patients underwent two echocardiographic assessments during the same visit, using the same scanner on the same bed and in the same room by two accredited echocardiographers (AW, JA) blinded to each other’s assessment. Scan-rescan reproducibility was determined for haemodynamic measures of stenosis severity (peak velocity, mean gradient, AVA and DI).

### Assessment of disease progression

Participants in the *disease progression cohort* returned for repeat clinical assessment and echocardiography at 1 and 2 years as well as repeat CT at either 1 or 2 years.^13^ The same scanner and imaging protocol was used for all CT scans and echocardiograms were performed by the same echocardiographer (AW) using the same scanner. To assess disease progression, annualised differences in each measure of stenosis severity were calculated. In participants who underwent three echocardiograms (baseline, 1 and 2 years), a line of best fit was used to determine annualised progression. Finally, Cohen’s d-statistic (*d*) was calculated for CT-AVC and each echocardiographic assessment (peak velocity, mean gradient, AVA, DI) to express the magnitude of progression normalised by the uncertainty in the measurement technique. This was calculated by dividing the magnitude of the annualised progression by the measurement repeatability, defined as (1/√2) of the SD of the differences between measurements at scan and rescan within the reproducibility cohort.^14^

### Group size analysis

Power analysis was performed to determine group sizes needed to detect changes in CT-AVC and echocardiographic parameters in a hypothetical clinical trial. To determine the group size needed to detect changes after a therapeutic intervention in a single group, power analyses were based on paired t-tests. The annualised progression and measurement repeatability for each modality were used to compute the effect size and subsequently group sizes required to detect i) disease progression using CT-AVC and each echocardiography measure and ii) sample sizes needed to detect treatment effects on disease progression using the different modalities. Treatment effects of 30%, 20% and 10% of the annualised progression values measured in the progression group were considered. Group sizes were estimated for powers of 70%, 80% and 90% and an error probability (α) of 0.05. Analysis was carried out using G-power software.^15^

### Statistical methods

Continuous variables were expressed as either mean±SD or median (IQR) depending on normality. Parametric (unpaired Student’s t-test) and non-parametric (Mann-Whitney U) tests were used for independent variables as appropriate. Categorical data were presented as n (%) and compared when appropriate using a contingency table and Fisher’s or χ^2^ tests. Reproducibility was assessed using Bland-Altman analysis and intraclass correlation (ICC). Correlation between continuous variables was assessed with linear regression analysis and either Pearson’s r or Spearman’s Rho subject to normality. Annualised rates of progression were calculated using the difference between two time-points (CT-AVC) or regression analysis over three time-points (echocardiography). Statistical significance was defined as two-sided p<0.05.

## Results

### Study population

Thirty-three participants comprised the reproducibility cohort (aged 71±8, 68% male). Eighteen participants underwent two echocardiograms (aged 70±8, 67% male, table 1) and 15 underwent two CT scans (aged 73±7, 67% male, table 1). A total of 81 participants were enrolled in the disease progression cohort (aged 72%±8%, 69% male, table 1). Of these, 71 underwent repeat echocardiography and 61 underwent repeat CT-AVC. Ten CT scans were excluded due to suboptimal image quality, leaving 51 included in the analysis. There was a high prevalence of cardiovascular risk factors, with the majority of patients having co-existing hypertension.

**Table 1:** Baseline clinical characteristics

	Reproducibility cohort		Disease progression cohort		
	Echocardiography	CT	All	Echocardiography follow-up	Calcium score follow-up
Number	18	15	81	71	51
Age (years)	70±8	73±7	72±8	72±8.2	73±7
Male	12 (67)	10 (67)	55 (69)	50 (70)	34 (67)
Body mass index (kg/m^2^)	30±4	30±6	29±5	28±4	28±4
Systolic blood pressure (mm Hg)	152±20	151±18	146±20	144±18	143±17
Comorbidity					
Diabetes mellitus	4 (22)	4 (27)	15 (18)	13 (18)	9 (18)
Hypertension	13 (72)	11 (73)	60 (74)	50 (70)	36 (70)
Documented CAD	9 (50)	6 (40)	33 (41)	31 (44)	26 (51)
Current smoker	8 (44)	6 (40)	9 (11)	8 (11)	5 (9)
Serum creatinine (mg/dL)	0.89±0.23	0.70±0.11	1.01±0.31	1.00±0.30	0.98±0.28
Medications					
ACE inhibitors	8 (44)	6 (40)	32 (40)	27 (38)	20 (39)
AIIRB	4 (22)	3 (20)	11 (13)	9 (13)	7 (13)
Beta-blockers	7 (39)	7 (47)	33 (41)	30 (43)	23 (45)
Statins	14 (78)	9 (60)	54 (66)	46 (64)	34 (67)
Baseline echocardiographic assessment					
AV jet velocity (m/s)	3.5 (3.2–4.0)	3.3 (3.0–3.8)	3.4 (2.8–4.1)	3.3 (2.7–3.9)	3.0 (2.5–3.6)
AV mean gradient (mm Hg)	25 (21–31)	24 (22–30)	25 (16–36)	24 (14–32)	21 (13–25)
AV area (cm^2^)	1.1 (0.8–1.3)	1.1 (1.0–1.3)	1.1 (0.9–1.4)	1.2 (0.9–1.5)	1.2 (1.0–1.5)
Dimensionless index	0.30 (0.23–0.37)	0.32 (0.25–0.40)	0.32 (0.25–0.39)	0.33 (0.26–0.39)	0.36 (0.30–0.40)
CT assessment					
AV calcium score (AU)	989 (497–1708)	1178 (579–2109)	1339 (553–2422)	1190 (505–2182)	874 (459–1792)

Mean±SD, median (IQR) and number (percentage).

AIIRB, angiotensin 2 receptor antagonists; AS, aortic stenosis; AU, Agatson Units; AVA, aortic valve area; CAD, coronary artery disease; LV, left ventricle; LVH, left ventricular hypertrophy.

### Reproducibility cohort

**Figure 1:**
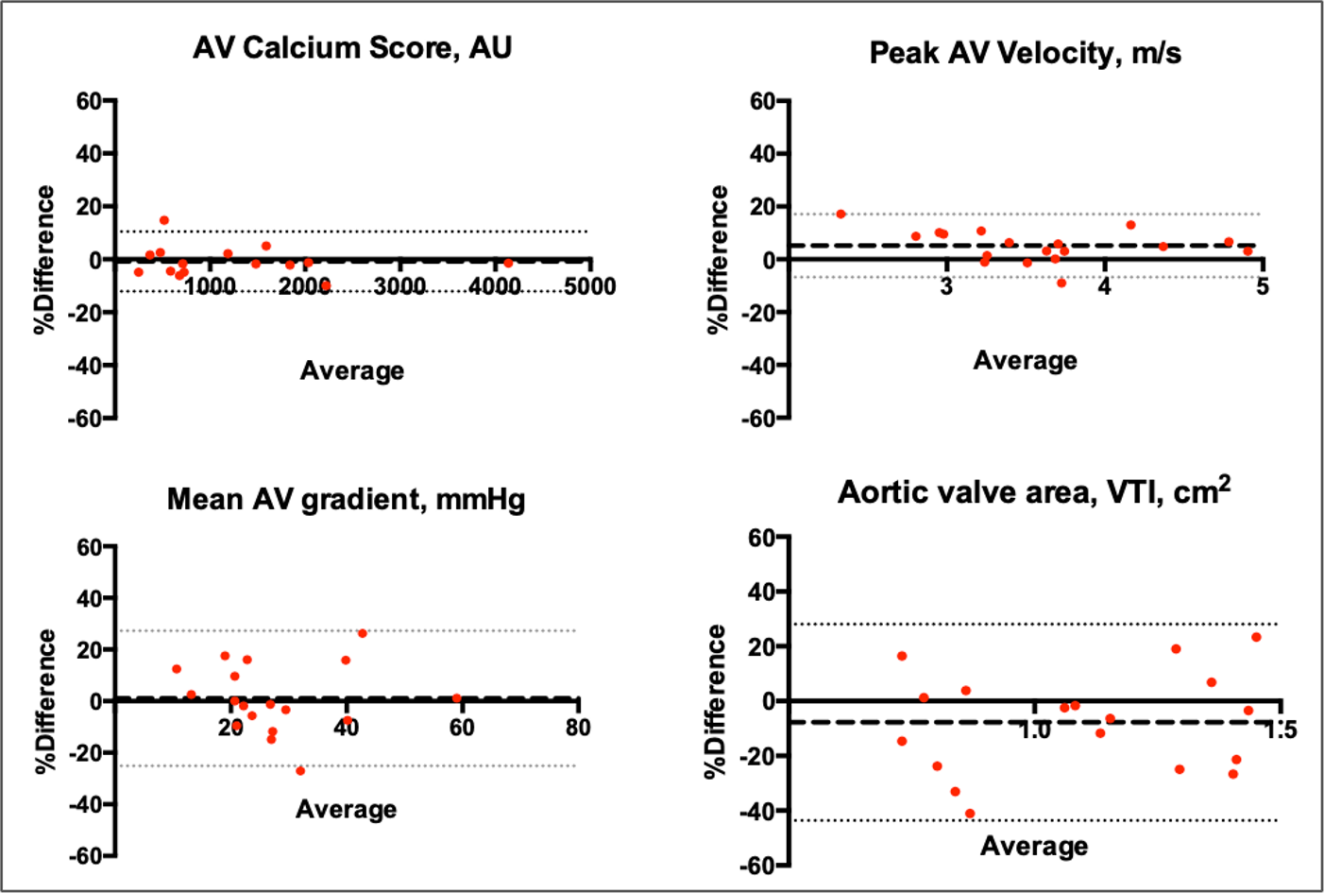
Scan-rescan reproducibility of CT quantification of aortic valve calcification (CT-AVC) and echocardiographic assessment of aortic stenosis severity. Bland-Altman plots displaying the scan-rescan reproducibility of aortic stenosis severity measurements on serial CT-AVC (3.9±3.3 weeks) and echocardiography (1±0 days). VTI, velocity time integral.

Within the reproducibility cohort (n=33), 15 patients underwent two non-contrast ECG-gated CT scans within 19 (IQR 14–28 days, range 7–98 days). Scan-rescan reproducibility for CT-AVC was excellent, without fixed or proportional bias (mean difference −1% (limits of agreement −12% to 10%), ICC 0.99; table 2, figure 1). Measurement variability for CT-AVC was 49 AU, or 4.2% when normalised to the median CT-AVC at baseline. Intraobserver (median CT-AVC 1178 AU, mean difference 1% (limits of agreement 9% to −11%), ICC 0.99) and interobserver (median CT-AVC 1207 AU, mean difference 0% (limits of agreement −5% to 6%), ICC 0.99) reproducibilities were also excellent. Scan-rescan reproducibility was also assessed with two different observers and demonstrated good reproducibility (ICC 1.00 (95% CI 0.99 to 1.00), mean difference 2.57% (44 AU) and limits of agreement −27.5% to 22.4%) (online supplemental figure S2).

**Table 2:** Reproducibility of CT-AVC and echocardiography assessments of aortic stenosis severity

	Bias, % (95% limits of agreement)	Bias, units (SD of difference of scan 1 and scan 2)	Measurement repeatability, units (%)	Intraclass correlation (95% CI)
CT-AVC				
Scan-scan reproducibility, AU	−1 (−12–10)	−20 (69)	49 (4.2%)	0.99 (0.99 to 1.00)
Intraobserver reproducibility, AU	−1 (−11–7)	−12 (85)	–	1.00 (1.00 to 1.00)
Interobserver reproducibility, AU	0 (−5–6)	11 (65)	–	0.99 (0.99 to 1.00)
Echocardiography, scan-rescan				
AV max, m/s	5 (−7–17)	0.17 (0.20)	0.14 (4.0%)	0.96 (0.78 to 0.99)
Mean gradient, mm Hg	1 (−25–27)	0.28 (4.3)	3.0 (12.0%)	0.97 (0.91 to 0.99)
AVA (VTI), cm^2^	−8 (−44–28)	−0.11 (0.24)	0.17 (15.5%)	0.85 (0.59 to 0.94)
Dimensionless index	−2 (−11–15)	0.01 (0.02)	0.01 (3.3%)	0.98 (0.95 to 0.99)

AU, Agatson Units; AVA, aortic valve area; CT-AVC, CT quantification of aortic valve calcification; VTI, velocity time integral.

Eighteen patients underwent two transthoracic echocardiograms during a single study visit. Scan-rescan reproducibility was excellent for peak velocity (mean difference 5%; limits of agreement −7% to 17%; ICC 0.96; measurement repeatability 4.0%; ICC 0.96) and DI (mean difference −1.7%; limits of agreement −11.1% to 14.5%; ICC 0.98; measurement repeatability 3.3%). Reproducibility was also good for mean gradient (mean difference 1%; limits of agreement −25% to 27%; ICC 0.97; measurement repeatability 12.0%; ICC 0.97) but less reliable in the assessment of AVA (mean difference −8%; limits of agreement −44% to 28%; ICC 0.85, measurement repeatability 15.5%) (table 2).

### AVC and aortic stenosis severity

At baseline, CT-AVC correlated with all echocardiographic measures (online supplemental table S1). The closest associations were observed with peak velocity (r=0.75 (95% CI 0.63 to 0.84), p<0.001) and mean gradient (r=0.75 (95% CI 0.64 to 0.83), p<0.001) and the weakest with AVA (r=−0.46 (95% CI −0.61 to −0.25), p<0.001).

### Patient follow-up

During the 2-year follow-up period (736 (IQR 722–760) days), 61 participants (75%) underwent repeat CT (13 at 1 year and 48 at 2 years). Images were non-interpretable in 10 participants (16%) due to motion artefact and were excluded, predominantly in those in whom beta-blockers were contraindicated. Follow-up echocardiography was performed in 71 participants at 1 year and 62 at 2 years. No echocardiography scans were excluded from analysis.

### Aortic stenosis disease progression

Across all patients, modest progression in each echocardiographic measure of severity was observed (Δ peak velocity 0.12 (IQR 0.0–0.25) m/s/year; Δ mean gradient 2.0 (IQR 0.0–4.0) mm Hg/year; Δ AVA −0.11 (IQR −0.26–0.02), Δ DI −0.02 (IQR −0.04 to −0.01)) (table 3). When patients were divided into mild, moderate and severe aortic stenosis, those in the moderate group demonstrated the clearest evidence of disease progression (n=30, Δ peak velocity 0.18 (IQR 0.06–0.30) m/s/year; Δ mean gradient 3.2 (IQR 0.7–5.4) mm Hg/year) (table 3).

**Table 3:** Disease progression on echocardiography and CT-AVC in patients with aortic stenosis

Variable	All patients	Mild aortic stenosis	Moderate aortic stenosis	Severe aortic stenosis
Baseline echocardiography				
No. of patients	81	25	33	23
Peak aortic jet velocity, (m/s)	3.4 (2.8–4.1)	2.5 (2.4–2.7)	3.4 (3.2–3.7)	4.5 (4.1–5.1)
Mean gradient (mm Hg)	25 (16–36)	13 (11–16)	25 (22–29)	43 (38–58)
Aortic valve area (cm^2^)	1.1 (0.9–1.4)	1.4 (1.2–1.7)	1.1 (1.0–1.3)	0.8 (0.6–0.9)
Dimensionless index	0.32 (0.25–0.39)	0.42 (0.38–0.49)	0.31 (0.28–0.33)	0.21 (0.20–0.25)
Follow-up echocardiography				
No. of patients	71	24	30	17
Δ aortic jet velocity (m/s/year)	0.1(0.0–0.3)	0.1 (0.0–0.2)	0.2 (0.1–0.3)	0.1 (−0.1–0.2)
Δ aortic jet velocity (%m/s/year)	3.5 (0.0–7.8)	3.2 (−0.7–6.3)	5.0 (2.3–10.2)	3.2 (−1.1–5.2)
Cohen’s d-statistic	0.71	0.71	1.43	0.71
Δ mean gradient (mm Hg/year)	2 (0–4)	1 (0–2)	3 (1–5)	3 (0–5)
Δ mean gradient (%mm Hg/year)	9.5 (−0.5–17.0)	7.5 (−2.4–14.9)	11.6 (2.4–29.5)	7.0 (−1.7–13.7)
Cohen’s d-statistic	0.66	0.33	1.0	1.0
Δ aortic valve area (cm^2^/year)	−0.1 (−0.2 to 0.0)	−0.1 (−0.2 to −0.0)	−0.1 (−0.1 to −0.0)	0.0 (−0.1 to −0.0)
Δ aortic valve area (%cm^2^/year)	−8.7 (−14.4 to −2.9)	−5.2 (−13.3 to −0.6)	−9.9 (−15.6 to −4.8)	−7.7 (−15.0 to 0)
Cohen’s d-statistic	0.59	0.59	0.59	0.0
Δ Dimensionless index	−0.02 (−0.04 to −0.01)	−0.02 (−0.04 to 0.00)	−0.02 (−0.04 to −0.01)	−0.01 (−0.02 to 0.00)
Δ Dimensionless index (%/year)	−5.7 (−11 to −2.0)	−4.4 (10.0 to −0.3)	−6.7 (−13.6 to −2.9)	−5.7 (−10.3 to 2.3)
Cohen’s d-statistic	1.41	1.41	1.41	0.71
Baseline CT				
No. of patients	72	23	30	19
AV calcium score (AU)	1339 (553–2422)	489 (281–693)	1427 (777–2215)	3386 (1770–6211)
Follow-up CT				
No. of patients	51	21	24	6
Δ AV calcium score (AU/year)	152 (65–375)	64 (48–134)	289 (106–443)	342 (163–583)
Δ AV calcium score (%AU/year)	20.0 (13.0–24.5)	20.3 (17.5–31.1)	20.0 (10.8–24.5)	16.9 (10.9–24.4)
Cohen’s d-statistic	3.12	1.30	5.90	6.98

AU, Agatson Units; AV, aortic valve; CT-AVC, CT quantification of aortic valve calcification.

**Figure 2:**
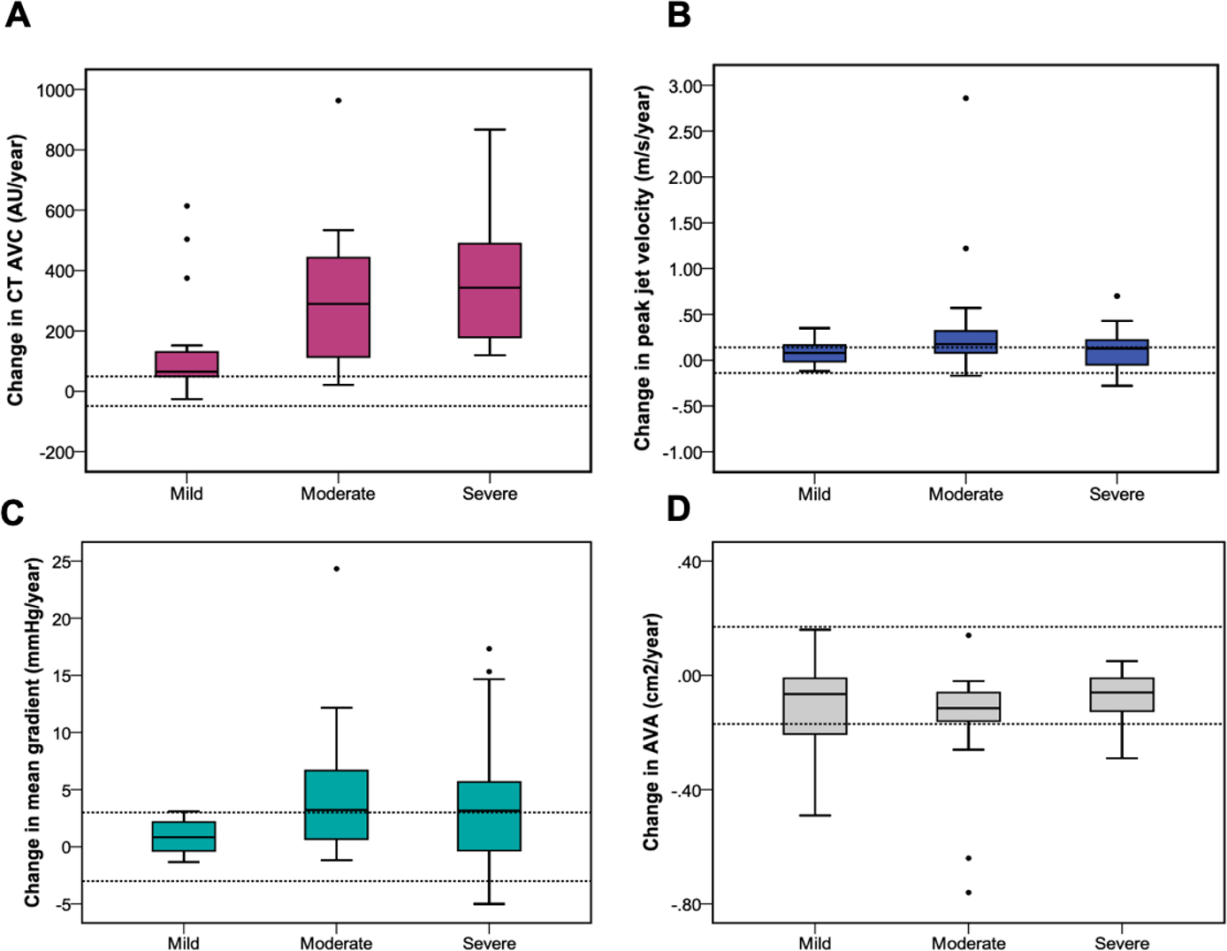
Aortic stenosis disease progression measured using CT quantification of aortic valve calcification (CT-AVC) and echocardiography. Annualised disease progression across each cohort using CT calcium scoring (A), peak aortic jet velocity (B), mean gradient (C) and aortic valve area (D). Relatively large annualised changes in the CT calcium score are observed compared with smaller changes and wide overlap in the measurements obtained by echocardiography. Dashed lines demonstrate the expected measurement repeatability from scan-rescan measurements.

Across the cohort as a whole, CT-AVC progressed by 152 (IQR 65–375) AU/year, with the most rapid rates of progression observed in participants with the most severe disease (Δ CT-AVC; mild AS 64 (IQR 48–134) AU/year, moderate AS 289 (IQR 106–443) AU/year, severe AS 342 (IQR 163–583) AU/year) (table 3, figure 2).

**Figure 3:**
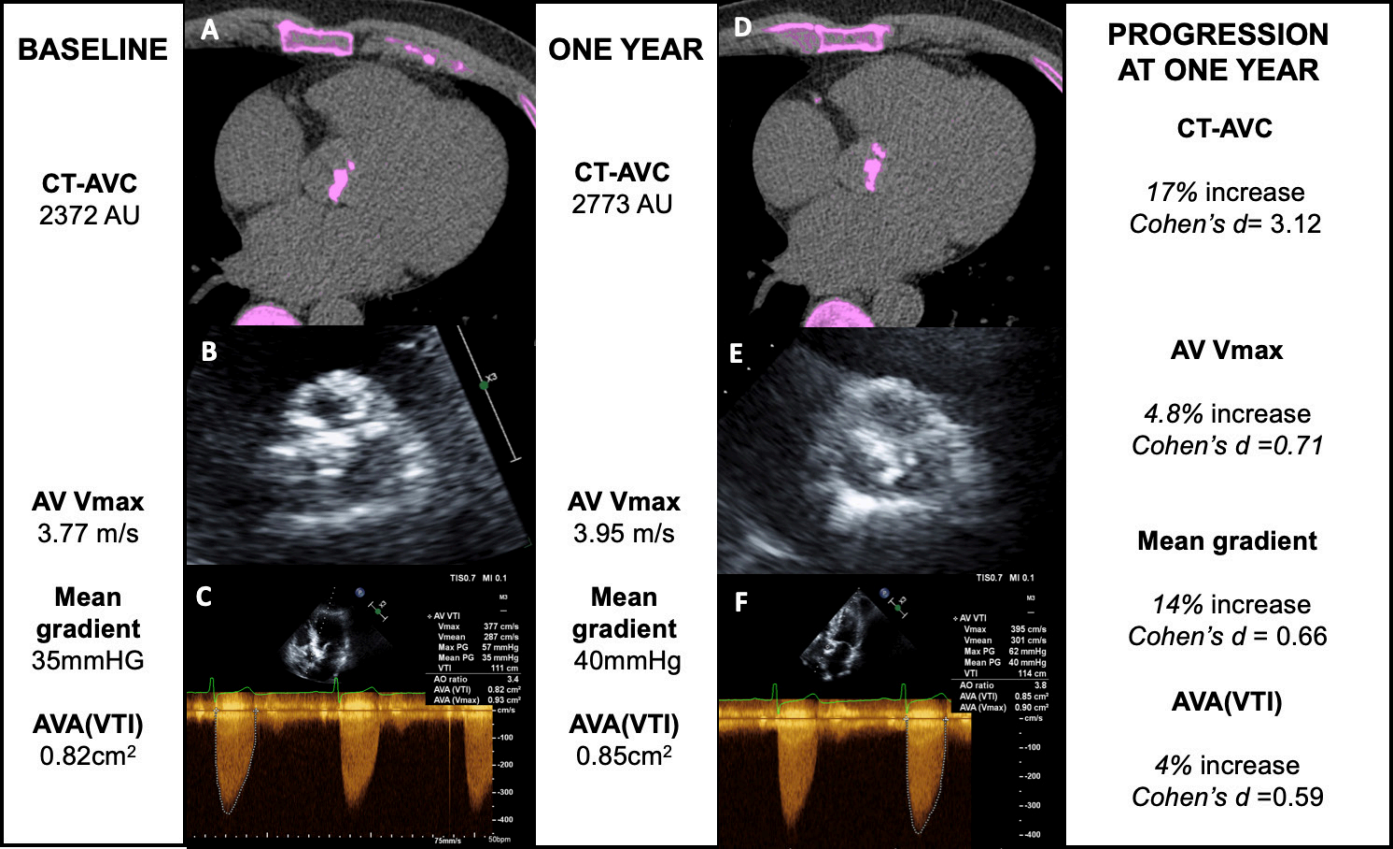
CT calcium scoring and echocardiography to monitor disease progression in aortic stenosis. CT calcium scoring of the aortic valve (AV) and echocardiography in a patient at baseline (A–C) and 1 year (D–F). Baseline CT calcium scoring demonstrates CT quantification of aortic valve calcification (CT-AVC) of 2372 AU (A), transthoracic echocardiography of the AV shows calcified leaflets with a calcium score of 4 (B) and Doppler echocardiography demonstrates a peak velocity of 3.77 m/s (C) at baseline. At 1 year, CT-AVC has increased to 2773 (D), the AV calcium score on echocardiography is graded as 4 (E) and the peak jet velocity has increased to 3.95 m/s (F). AVA, aortic valve area; VTI, velocity time integral.

The Cohen’s d-statistic (*d*) was calculated by dividing the overall annualised rate of change in each severity measure by the measurement repeatability (table 3) By this method, CT-AVC displayed a greater progression to measurement repeatability ratio (CT-AVC: *d*=3.12) when compared with each echocardiographic parameter (peak velocity: *d*=0.71; mean gradient: *d*=0.66; AVA: *d*=0.59; DI *d*=1.41). When patients with more advanced disease were considered, the differences between echocardiography and CT-AVC were greater. In participants with severe aortic stenosis, CT-AVC displayed a greater than sixfold higher value (*d*=6.98) compared with echocardiographic measures (peak velocity *d*=0.71; mean gradient *d*=1.0; AVA *d*=0.0; DI *d*=0.71). In those with moderate and mild disease, the d-statistic for CT-AVC remained higher when compared with echocardiographic measures of the same severity (table 3, figure 3).

### Group size analysis

**Figure 4:**
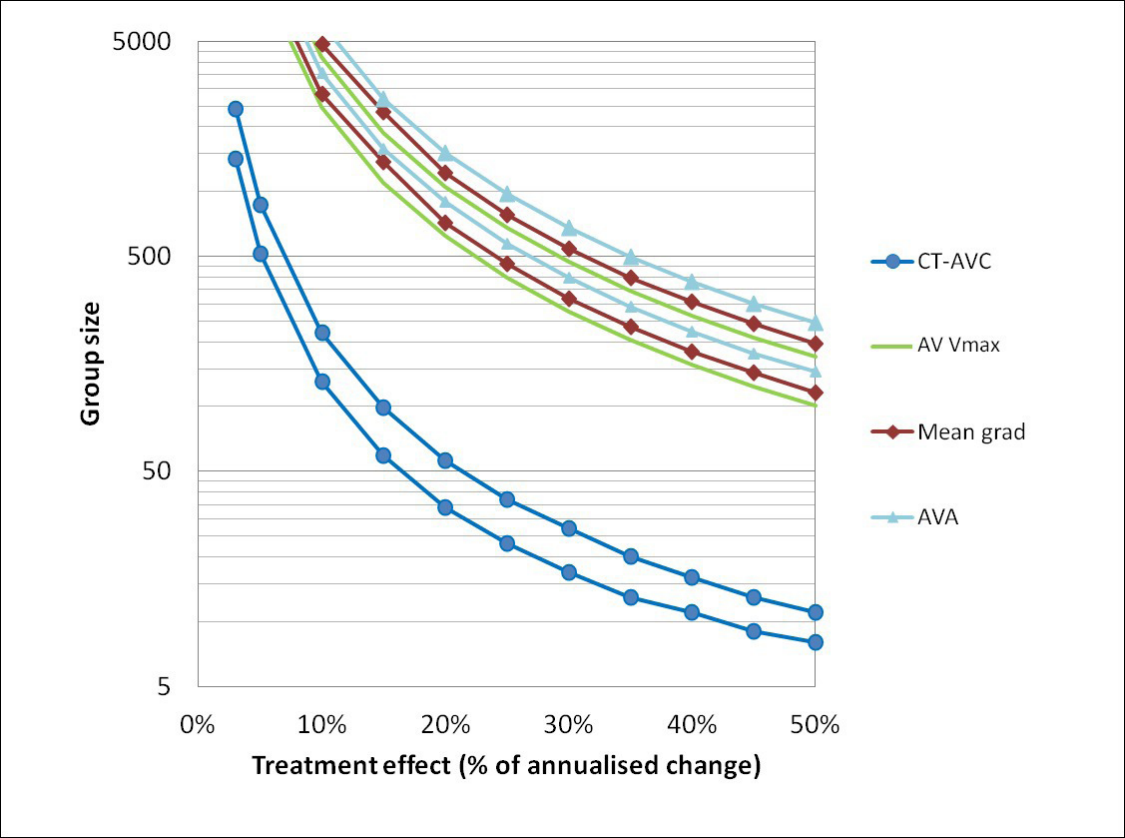
Sample sizes needed for studies of novel therapies in aortic stenosis using CT quantification of aortic valve calcification (CT-AVC) to assess their effect on disease progression. The number of participants required in a study to detect a given treatment effect size at different levels of power are plotted. For each modality an upper bound at 90% power and lower bound at 70% are plotted with α=0.05 for all. Nominal treatment effects up to 50% of the measured annualised progression for each modality are considered. Group size calculations should also consider the proportion of non-interpretable scans that may be encountered. AVA, aortic valve area.

Using the cohort-averaged progression and measurement repeatability values, the effect size for a matched-pairs t-test was computed for CT-AVC and each echocardiographic measure. The group size required to detect annualised disease progression for CT-AVC was smaller than for echocardiographic measures (CT-AVC: 4, peak velocity: 33, mean gradient: 39, AVA: 48 (α=0.05, power=80%)). Similarly, the group size required to detect a treatment effect of a new therapy (30%, 20% and 10% reduction in the annualised progression) was >10-fold smaller for CT-AVC than for echocardiographic measures (CT-AVC: 20, 43, 165 patients, respectively; peak velocity: 351, 787, 3142 patients, respectively; mean gradient: 403, 910, 3632 patients, respectively; AVA: 505, 1134, 4516 patients, respectively (α=0.05, power=80%)) (figure 4). Group size calculations should also consider the proportion of non-interpretable scans that may be encountered.

## Discussion

In this study, we have investigated the utility of CT-AVC and echocardiography in assessing disease progression in patients with aortic stenosis. CT-AVC demonstrated excellent scan-rescan reproducibility. Calculation of Cohen’s d-statistic demonstrated that CT-AVC had a large annualised change normalised to measurement repeatability compared with echocardiography, with a fourfold higher value for CT-AVC (*d*=3.12) compared with peak velocity (*d*=0.71). (figure 3) CT-AVC may therefore be a useful technique both in clinical practice for assessing aortic stenosis progression and as an endpoint in clinical trials assessing the efficacy of novel therapies.

Given the central role of calcification in the progression of aortic stenosis, direct assessments of calcification hold major interest.^16^ Quantification of aortic valve calcification has demonstrated considerable promise in a number of studies, discerning the presence of severe stenosis among patients with discordant echocardiography and providing powerful prognostic information.^5 17 18^ In this study, we have demonstrated excellent scan-rescan reproducibility of CT-AVC coupled with relatively large progression in values over time, demonstrating the feasibility and potential advantages of this modality for tracking aortic stenosis progression.

Before an imaging technique can be applied to routine clinical practice, clinicians must be reassured that the technique is robust and reproducible. Measurement of CT-AVC is technically straightforward, although can be complicated by motion artefact, particularly in patients with advanced disease, systolic dysfunction or conduction disease in whom administration of beta-blockade is not possible. In this study, this resulted in exclusion of 16% of scans. This limitation of CT-AVC must be considered when deciding whether to use this test in the clinical or research settings and certain patient characteristics including body mass index, tachycardia or contraindications to beta-blockade may be used to identify patients in whom CT-AVC may be unreliable. However, when CT-AVC is possible we have demonstrated that excellent reproducibility can be achieved with a consistent and standardised approach. While all echocardiography scans were of diagnostic quality in our study, we acknowledge that there is a selection bias in our patient population since participants were only recruited if they had clear assessments of aortic stenosis severity on clinical echocardiograms. In non-selective cohorts, it is estimated that 10%–15% of patients have poor echocardiographic windows, similar to our observations for CT-AVC. Furthermore, variability in echocardiography was minimised by performing two echocardiograms under as consistent conditions as possible, thereby diminishing many sources of error encountered in clinical practice. This approach necessitated two different echocardiographers performing the scan to avoid recall bias.

For echocardiography, while peak velocity and mean gradient both demonstrated good reproducibility, repeatability of AVA was poor, likely reflecting the multiple different measurements required for its calculation. Measurement of the LVOT diameter represents the major source of error in calculation of AVA. By disregarding LVOT diameter and considering a simplified ratio of LVOT-to-aortic velocity, the DI overcomes discrepancies in LVOT measurements and has proven effective in distinguishing aortic stenosis severity and predicting adverse outcomes.^19–21^ We have shown that this measure improves reproducibility when compared with AVA.

In order to compare the utility of CT-AVC and echocardiography for tracking disease progression, both the magnitude of change and measurement repeatability must be considered. On this basis, we calculated the Cohen’s d-statistic for each modality. This indicated that CT-AVC appears superior to echocardiography for detecting small changes in disease severity over time. Indeed, this marker was four times higher for CT-AVC compared with peak velocity. This may have important clinical implications when tracking disease progression and predicting when patients are likely to require valve intervention. Further research is required to assess how this approach may work in clinical practice, but CT-AVC is likely to be of particular value in patients whose heart rate can be optimised for imaging and who have poor echocardiographic windows or low flow states where mean gradient and peak velocity may underestimate disease severity.

Our data also support a role for CT-AVC as an end point in research trials investigating the effects of novel therapies. Imaging end points are increasingly adopted for this purpose, and those with greater reproducibility and sensitivity to detect small changes in progression are likely to minimise cost and sample sizes required (SALTIRE 2 NCT02132026). Indeed, our data suggest that >10-fold fewer patients would be required to detect 10%, 20% and 30% treatment effects using CT-AVC (165, 43 and 20 patients, respectively) compared with peak velocity (3142, 787 and 351 patients, respectively). While this suggests a considerable advantage in using CT-AVC, consideration should be placed on the frequency of non-diagnostic scans and clinical characteristics for which CT-AVC may not be suitable. Indeed, the proportion of non-interpretable CT scans must be taken into account when considering sample sizes and measurement repeatability. It is also important to consider that CT-AVC does not account for the effects of therapy on non-calcific valve thickening (ie, fibrosis) and echocardiography remains the first-line imaging technique to assess aortic stenosis. Furthermore, another important consideration is the radiation dose associated with CT, although standard imaging techniques enable CT calcium scoring to be performed with low levels of radiation exposure (1–3 mSV).^22^

Our study has several limitations. We acknowledge that our study numbers are small and in the reproducibility cohort, different patients underwent repeat CT-AVC to those undergoing repeat echocardiography. While the characteristics of both patient groups were similar, we cannot rule out the possibility of confounding variables and recognise this as a limitation of our study. Our study findings should therefore be confirmed in larger studies with direct comparisons of reproducibility and disease progression for echo and CT-AVC within the same population. Furthermore, we assessed annualised changes in disease severity in all patients who underwent follow-up beyond 1 year. This assumes linear disease progression and therefore does not take into account a more rapid rise in disease progression as severity increases, although this is consistent with other studies investigating disease progression in aortic stenosis.^23 24^

## Conclusion

CT-AVC is a robust and reproducible imaging technique that holds major promise as a method for tracking disease progression in aortic stenosis.

**Key questions**

**What is already known on this subject?**

- Aortic stenosis remains an important cause of morbidity and mortality worldwide and its burden is set to increase.
- Two-dimensional echocardiography is currently the gold standard imaging modality to define and track changes in disease severity over time.
- CT has recently emerged as a useful modality to quantify valvular calcification burden in aortic stenosis and provides prognostic information.

**What might this study add?**

- In this study, we have highlighted that measurement of aortic valve calcification by CT is reproducible and may be valuable in detecting small changes in disease severity over time, potentially highlighting patients with more rapid progression of aortic stenosis.

**How might this impact on clinical practice?**

- By providing a sensitive measure of disease progression, the measurement of aortic valve calcification by CT may be useful in both highlighting patients with rapidly progressive aortic stenosis and as an end point in clinical trials of novel therapies designed to slow disease progression.
