# Supplementary material for: Computed tomography aortic valve calcium scoring for the assessment of aortic stenosis progression
Source: Heart. 2020 Oct 5;106(24):1906–13. doi: 10.1136/heartjnl-2020-317125 (PMC7719911; doi:10.1136/heartjnl-2020-317125)
Supplement: Supplementary data [file heartjnl-2020-317125supp001.pdf]

Supplemental Data

Supplemental Figure S1. Computed Tomography Calcium Scoring.

Computed Tomography calcium scoring performed on contiguous slices of the aortic valve in the axial plane. Examples from patients with mild (A) and severe (B) aortic stenosis are shown. All calcium is highlighted in pink. Contours are then drawn around calcium in the aortic valve which then turns yellow once selected. An overall score in Arbitrary units and a calcium volume are then generated.

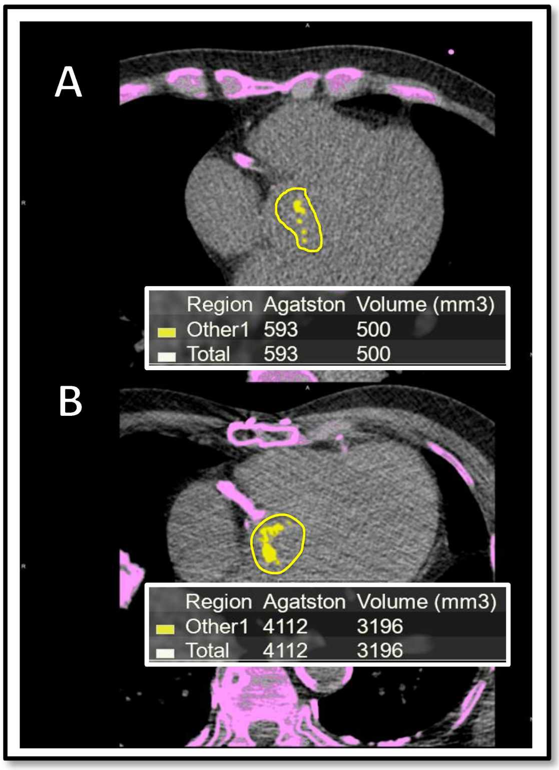

Supplemental Figure S2. Scan-rescan reproducibility of CT-AVC using a different observer for each scan.

Scan-rescan reproducibility was also assessed with two different observers and demonstrated good reproducibility (ICC 1.00 (0.99-1.00) mean difference 2.57% (44 AU) and limits of agreement -27.5-22.4%.

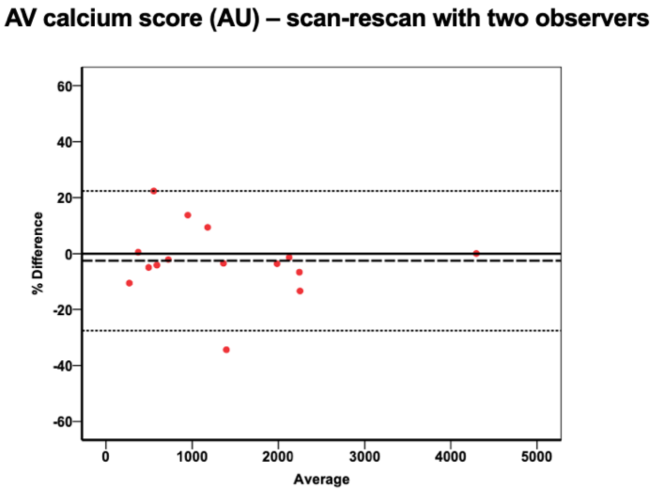

Supplemental Figure S3. Comparison of annualised progression measured by echocardiography over two time points or three time points using regression analysis.

Variable	Annualised Change over 2 time points	Annualised change over 3 time points	P value
Δ aortic jet velocity (m/s/year)	0.12 [0.00-0.25]	0.12 [0.00-0.27]	0.87
Δ mean gradient (mmHg/year)	2.00 [-0.05-4.00]	2.00 [-0.05-4.00]	0.66
Δ aortic-valve area (cm^2/year)	-0.11 [-0.26 to -0.02]	-0.10 [-0.16 to -0.03]	**<0.001**

Data represents median [interquartile range].

Supplemental Table S1.
Pearson's correlations between square-root transformed computed tomography calcium scores (AVC and AVCd) with echocardiographic indices. R values (95% confidence intervals) are shown.

	**CT-AVC (AU)**	**AVCd (AU/cm^2)**	**Peak Aortic Jet Velocity (m/s)**	**Mean Aortic Gradient (mmHg)**	**Aortic Valve Area (cm^2)**
**CT-AVC (AU)**		0.98 (0.97 to 0.99) P<0.001	0.75 (0.63 to 0.84) P<0.001	0.75 (0.64 to 0.83) P<0.001	-0.46 (-0.61 to -0.25) P<0.001
**Peak Aortic Jet Velocity (m/s)**	0.75 (0.63 to 0.84) P<0.001	0.78 (0.66 to 0.86) P<0.001		0.97 (0.96 to 0.98) P<0.001	-0.66 (-0.76 to -0.53) P<0.001
**Mean Aortic Gradient (mmHg)**	0.75 (0.64 to 0.83) P<0.001	0.78 (0.69 to 0.86) P<0.001	0.97 (0.96 to 0.98) P<0.001		-0.71 (-0.79 to -0.59) P<0.001
**Aortic Valve Area (cm^2)**	-0.46 (-0.61 to 0.25) P<0.001	-0.56 (-0.69 to -0.38) P<0.001	-0.66 (-0.76 to -0.53) P<0.001	-0.71 (-0.79 - -0.59) P<0.001	

Abbreviations: AVC, aortic valve calcium; AVCd, aortic valve calcium density; CE, continuity equation.
